# Supplementary material for: The causal role of circulating inflammatory markers in osteoporosis: a bidirectional Mendelian randomized study
Source: Front Immunol. 2024 Jul 18;15:1412298. doi: 10.3389/fimmu.2024.1412298 (PMC11291241; doi:10.3389/fimmu.2024.1412298)
Supplement: Supplementary file 1 [file DataSheet_1.zip › supplementary material/CIM-OSTEOPOROSIS /GCST90274821.csv_forest.pdf]

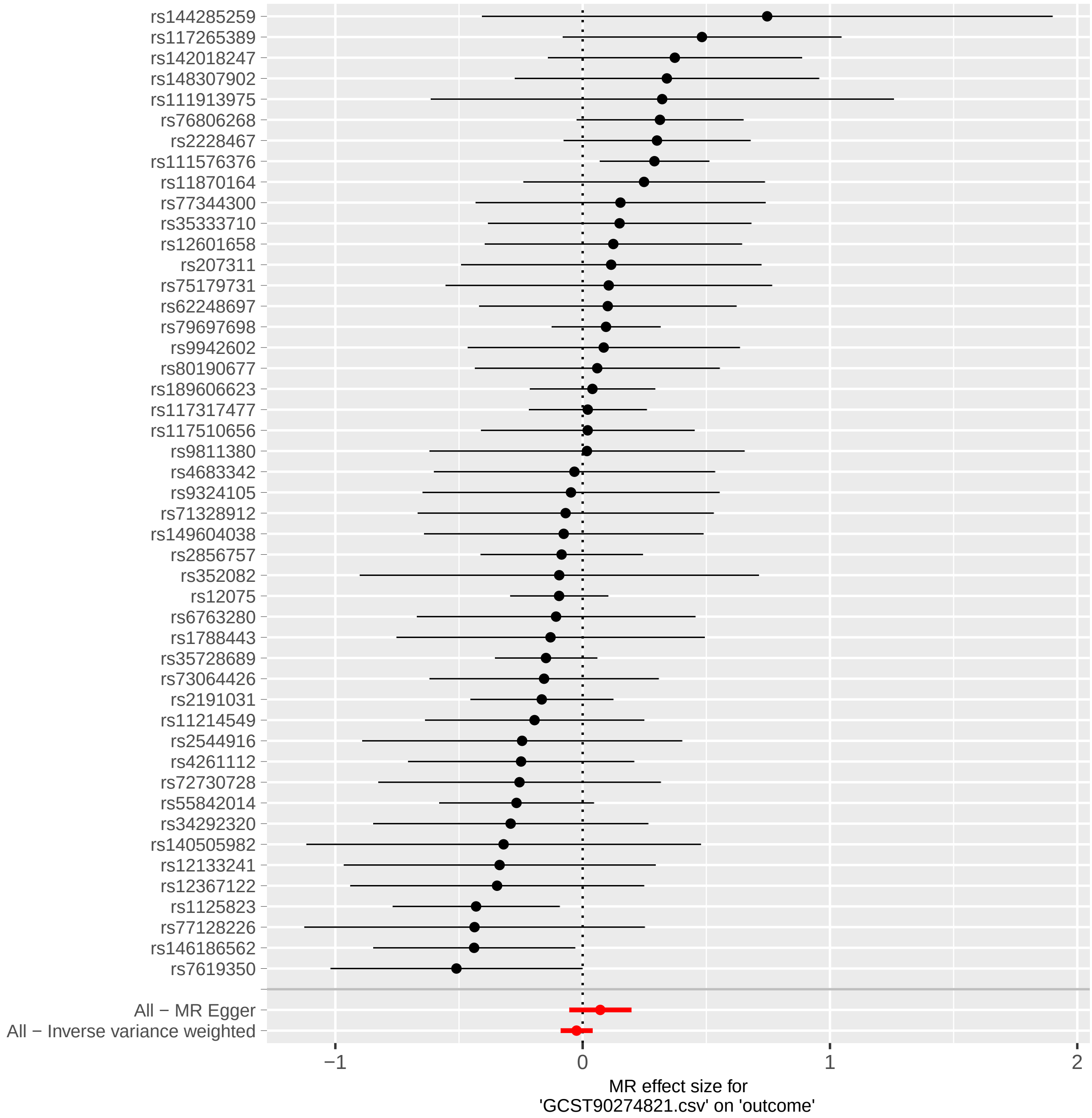
rs144285259
rs117265389
rs142018247
rs148307902
rs111913975
rs76806268
rs2228467
rs111576376
rs11870164
rs77344300
rs35333710
rs12601658
rs207311
rs75179731
rs62248697
rs79697698
rs9942602
rs80190677
rs189606623
rs117317477
rs117510656
rs9811380
rs4683342
rs9324105
rs71328912
rs149604038
rs2856757
rs352082
rs12075
rs6763280
rs1788443
rs35728689
rs73064426
rs2191031
rs11214549
rs2544916
rs4261112
rs72730728
rs55842014
rs34292320
rs140505982
rs12133241
rs12367122
rs1125823
rs77128226
rs146186562
rs7619350
All − MR Egger
All − Inverse variance weighted
−1
0
1
2
MR effect size for
'GCST90274821.csv' on 'outcome'
